# Clinical Phenotyping in Acute Respiratory Distress Syndrome: Steps Towards Personalized Medicine

**DOI:** 10.3390/jcm14207204

**Published:** 2025-10-13

**Authors:** Paul Leon Petrick, Martin Mirus, Lars Heubner, Hani Harb, Mario Menk, Peter Markus Spieth

**Affiliations:** 1 Department of Anesthesiology and Intensive Care Medicine, Faculty of Medicine and University Hospital Carl Gustav Carus, TUD Dresden University of Technology, 01307 Dresden, Germany; paulleon.petrick@ukdd.de (P.L.P.); martin.mirus@ukdd.de (M.M.); lars.heubner@ukdd.de (L.H.); mario.menk@ukdd.de (M.M.); 2 Institute of Medical Microbiology and Virology, Faculty of Medicine and University Hospital Carl Gustav Carus, TUD Dresden University of Technology, 01307 Dresden, Germany; hani.harb@ukdd.de

**Keywords:** acute respiratory distress syndrome, phenotyping, subphenotypes, treatable traits, multi-omics, precision medicine

## Abstract

Acute respiratory distress syndrome (ARDS) is a highly heterogeneous syndrome with a continuing high mortality rate. Despite intensive research, established therapies consist mainly of supportive measures, while pharmacological approaches have not yet shown any consistent survival benefits. In recent years, it has become clear that the great clinical and biological diversity of ARDS contributes significantly to the difficulty of demonstrating therapeutic effects. The phenotyping of ARDS has therefore become a central field of research. Different approaches—from clinical parameters and imaging to inflammatory and cardiovascular profiles and multi-omics analyses—have repeatedly identified reproducible subphenotypes that differ in prognosis and, in some cases, in response to therapies. Hypo- and hyperinflammatory subphenotypes have been described as particularly consistent. These are prognostically relevant and, in retrospective analyses, have also shown a differentiated response to glucocorticoids, statins, or fluid strategies. However, endotypes based on causal pathophysiological mechanisms are still largely theoretical. The concept of treatable traits illustrates the potential of personalized therapy but is currently based predominantly on retrospective findings. Future studies should use standardized terminology and multimodal approaches, take longitudinal data into account, and aim for prospective validation to define robust subphenotypes and causal endotypes. This could lay the foundation for true precision medicine in ARDS.

## 1. Introduction

### 1.1. One Syndrome, High Heterogeneity: The Need for ARDS Phenotyping and Conceptual Clarity

Acute respiratory distress syndrome (ARDS) is a severe and complex clinical condition characterized by acute hypoxic respiratory failure, bilateral pulmonary infiltrates, impaired oxygenation, and increased alveolar–capillary permeability [1]. In contemporary cohorts, ARDS is associated with mortality of approximately 35–45%, with the highest rates observed in severe cases [2]. Beyond acute mortality, ARDS survivors frequently experience sustained decrements in health-related quality of life—with persistent physical deconditioning, neurocognitive deficits, and psychological sequelae that can limit functional recovery for months to years—alongside prolonged ventilation and substantial intensive-care resource utilization [3,4]. Current best practice focuses on limiting ventilator-induced lung injury (low tidal volumes, constrained plateau/driving pressures), applying prone positioning, and avoiding fluid overload; however, putative anti-inflammatory, endothelial-stabilizing, or antithrombotic agents have largely failed to deliver reproducible survival benefits at the population level—suggesting that efficacy is phenotype-dependent and easily obscured in heterogeneous cohorts [5]. Extracorporeal membrane oxygenation (ECMO) may be considered in carefully selected patients with severe, refractory ARDS who fail to respond to conventional therapies, offering temporary pulmonary support while the underlying lung injury recovers. Recent studies and guidelines support the use of ECMO only in severe ARDS, highlighting its role as a rescue therapy in experienced centers for patients unresponsive to optimal conventional therapy [6,7]. This emphasizes the limitations of standard therapy for this highly heterogeneous syndrome. ARDS can result from a wide range of direct lung insults, such as pneumonia, aspiration of gastric contents, or inhalational injury, as well as indirect causes like sepsis, severe trauma, pancreatitis, or massive transfusion, all of which trigger a systemic inflammatory response leading to diffuse alveolar damage and impaired gas exchange. ARDS exhibits heterogeneity across multiple domains, including different causes (pulmonary vs. extrapulmonary), variability in the immune and inflammatory response, comorbidities, the temporal dynamics of disease onset, systemic organ failure, and a coagulopathy spectrum ranging from endotheliopathy-mediated hypercoagulability with microvascular thrombosis and fibrinolysis shutdown to consumptive or therapy-related hypocoagulability with heightened bleeding risk [8,9]. This variability complicates both prognostic assessment and the demonstration of therapeutic efficacy in randomized studies. In this context, ARDS phenotyping has gained importance, aiming to identify patient subgroups with shared clinical or biological characteristics to improve risk stratification, advance understanding of pathophysiological mechanisms, and ultimately enable precision medicine therapy [10]. Several methodological approaches have been applied to retrospectively identify such subgroups, including latent class analysis (LCA), clustering methods, and multi-omics profiling. These methods have revealed reproducible phenotypes with both prognostic and potentially therapeutic relevance. However, inconsistent terminology complicates the field. For example, terms such as subgroup, subphenotype, endotype, and treatable trait are often used interchangeably, despite reflecting different theoretical concepts. This conceptual inconsistency hampers comparability across studies and highlights the need for future efforts to develop an internationally consistent nomenclature.

The aim of this review is to provide a comprehensive summary of the current evidence on the phenotyping of ARDS. Different approaches, from clinical and inflammatory to cardiovascular and multi-omics-based phenotyping, as well as the associated methodological challenges, limitations, and implications for precision medicine and the design of clinical studies, are presented. This review aims to provide clarity on terminology and offer an overview of perspectives for future research that could ultimately contribute to personalized therapeutic approaches for ARDS.

### 1.2. Definitions and Terminology in ARDS Phenotyping

**Figure 1:**
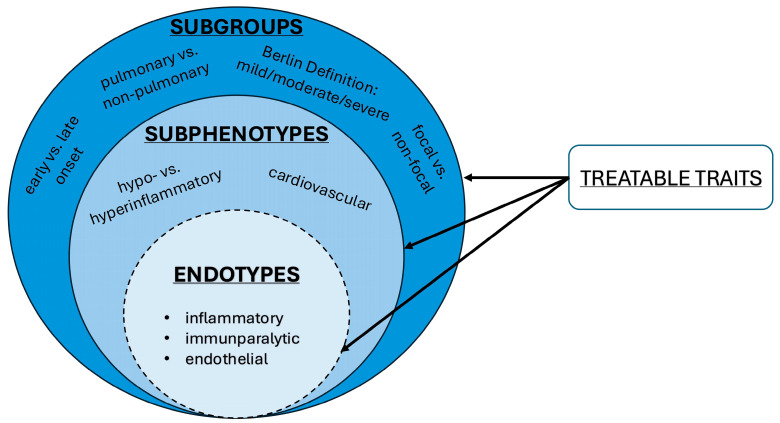
Conceptual framework of ARDS stratification. The arrows originating from “treatable traits” highlight that specific therapeutic targets or modifiable characteristics could be identified and addressed at each level of stratification.

Several terms are used in the literature to categorize ARDS patients (Figure 1), some of which are synonymous. The most important terms are explained in more detail below.

A phenotype describes the observable and measurable clinical characteristics of a patient, including physiology, laboratory results, and imaging findings, which emerge from the interaction of genetic factors, comorbidities, environmental influences, and therapeutic interventions. In ARDS, phenotypes encompass variables such as hypoxemia severity, lung mechanics, radiologic patterns, biomarker profiles, and coagulation status, and they may evolve dynamically over time [8,11,12]. ARDS itself is a phenotype.

By applying cut-off values, subgroups can be created within a phenotype [8,11,12]. The subdivision does not necessarily rely on biological markers; clinical variables can serve as the basis. A prominent example is the classification of ARDS severity in the Berlin definition [1].

When reproducible patterns of such features are identified and linked to outcomes or treatment responses, they can be classified into distinct subphenotypes. A subphenotype is defined as a distinct subgroup within a common phenotype, characterized by the expression of several shared traits, and distinguished from other subphenotypes [8,11,13]. In contrast to subgroups, which are usually based on clinically visible characteristics, subphenotypes describe deeper, often biologically determined characteristics within the heterogeneous entity of ARDS. Subphenotypes result from patterns in clinical, laboratory, and molecular biological data. Also, subphenotypes should be reproducible in different populations [12].

The term endotype has also been introduced in the literature [8,14,15]. Endotypes are defined by a particular, shared pathophysiological mechanism. An endotype represents a subphenotype that reacts differently to a targeted therapy. In other words, while a subphenotype is identified by observable clinical or biological patterns, an endotype goes one step further by linking these patterns to a specific causal pathway that can be selectively modified by treatment. Finally, the concept of a treatable trait will be discussed. This is a quantifiable, manageable, and medically beneficial characteristic of the patient, which has been shown to positively impact their outcome [11,13]. In contrast to phenotyping, the focus here is less on creating a homogeneous group and more on finding a targeted therapy decision. Subphenotypes and treatable traits may also be complementary.

## 2. Methods

### 2.1. Literature Search

A literature search was conducted on PubMed to identify clinical studies addressing phenotyping in ARDS patients. The search strategy included the following terms: (ARDS) AND (phenotype OR subphenotype OR endotype OR treatable trait OR subgroup). The titles and abstracts of the retrieved articles were screened for relevance. Studies were excluded if they did not provide primary data, focused on pediatric populations, reported non-clinical or animal data, or were not published in English. To enhance completeness and capture related publications beyond the initial PubMed results, Research Rabbit [16] was used to identify articles connected to the key studies.

### 2.2. Statistical Approaches to ARDS Phenotyping

Various methods have been used in research to identify subphenotypes in ARDS (Table 1), ranging from classic statistical approaches such as LCA to modern, data-driven machine learning techniques. These include clustering methods from the field of machine learning, examples of which are k-means or hierarchical clustering [17]. Such unsupervised methods operate without predefined groups and detect patterns directly in the data, for example, by automatically grouping patients with similar inflammatory profiles. LCA offers a slightly different, model-based approach [18]. LCA originates from classical statistics and is based on a probabilistic model that allows the membership of a subtype to be specified as a probability. LCA typically refers to a single point in time (e.g., admission or diagnosis). More advanced methods of classical statistics, such as growth mixture modeling or latent class growth analysis, enable the modeling of temporal progress [19], but have rarely been used in the context of ARDS.

Phenotyping requires prior selection of suitable parameters. In classical statistical methods, the selection is often theory-driven or based on clinical experience or previous studies.

In contrast, machine learning methods often automate the selection of relevant characteristics. Methods such as least absolute shrinkage and selection operator (LASSO) regression or random forest-based importance analyses are commonly applied [20]. These algorithms evaluate which variables contribute particularly well to the separation or grouping of patients while discarding irrelevant or redundant features.

After subphenotypes have been identified via LCA or clustering, many studies address how new patients can be accurately assigned to these subphenotypes in real time. Supervised machine learning is used for this purpose [21]. Algorithms such as random forest, support vector machine (SVM), or logistic regression model are trained on the original subphenotypes to enable robust classification using a minimal set of easily measurable variables. In ARDS research, predictive models based on only three to five parameters have been developed, yet still achieve high accuracy in subtypes assignment [22].

## 3. Subgroups of ARDS

ARDS is a syndrome that is caused by many factors. These factors suggest the possibility of subgroup formation. A rough subdivision can be made into pulmonary and non-pulmonary triggers [23,24]. Pulmonary triggers include pneumonia, aspiration, inhalation, and mechanical trauma (lung contusion). Sepsis is the most common extrapulmonary cause of ARDS [2] and defines a distinct subgroup with particularly high mortality, as demonstrated by Chang et al., who reported a 56% fatality rate in sepsis-associated ARDS [25].

Further stratification can be achieved based on the morphological characteristics, which can be visualized primarily by imaging procedures (computer tomographs, lung ultrasound, and electrical impedance tomography (EIT)). A distinction can be made between focal and non-focal patterns [26,27]. Focal describes a limited, usually caudal, lobar ventilation disorder. Patients with focal patterns often respond less well to recruitment maneuvers and benefit less from an aggressive setting of positive end-expiratory pressure (PEEP). In contrast, patients with diffuse infiltration, which is more common in non-pulmonary ARDS, have better recruitability [26]. EIT, as a functional imaging device, has been reported to detect differences between ARDS patients based on ventilation-perfusion mismatch [28].

A further possibility for subgroup formation is the categorization into early-onset ARDS and late-onset ARDS [29,30]. Early-onset ARDS is the development of ARDS within the first 48 h after hospital admission. Late-onset ARDS refers to cases after this time window. Late-onset ARDS often occurs in the context of prolonged intensive care courses with secondary infections or immunological dysregulation [31]. A poorer outcome has been shown for late-onset ARDS [31].

The most common subgrouping of ARDS patients is based on severity according to the Berlin definition [1]. The PaO_2_/FiO_2_ ratio is used to categorize patients into mild (201–300 mmHg), moderate (101–200 mmHg), and severe ARDS (<101 mmHg) groups. Many studies have shown that ARDS severity is an independent risk factor for the outcome of ARDS patients [32,33].

## 4. Subphenotypes of ARDS

### 4.1. Inflammatory Subphenotypes

The first subphenotyping of ARDS patients was carried out by Calfee et al. in 2014 [34]. Two subphenotypes were described. Subphenotype 1 (hypoinflammatory) was characterized by lower plasma concentrations of interleukin (IL)-6 and IL-8, plasminogen activator inhibitor-1, and by lower heart rates, whereas subphenotype 2 (hyperinflammatory) showed markedly elevated levels of these biomarkers, more severe organ dysfunction, and higher mortality. The categorization of patients to these subphenotypes was associated with significant differences in 90-day mortality across two ARDS cohorts: ARMA cohort [35]—subphenotype 1: 23%; subphenotype 2: 44%; *p* = 0.006; ALVEOLI cohort [36]—subphenotype 1: 19%; subphenotype 2: 51%; *p* < 0.001.

The aforementioned evidence of two inflammatory subphenotypes has been demonstrated in various other studies [37,38,39,40,41] and supplemented by additional parameters such as IL-18 [42] or soluble ST2-receptor [43,44]. Sathe et al. identified hypo- and hyperinflammatory subphenotypes via alveolar biomarkers [45]. However, classification based on plasma and alveolar biomarkers did not align, and no significant difference in 28-day mortality was observed for the hyperinflammatory subphenotype defined on alveolar biomarkers (28% vs. 13%, *p* = 0.12). ARDS has also been classified into uninflamed and reactive subphenotypes [46]. The reactive subphenotype, analogous to the hyperinflammatory type, is also characterized by elevated levels of biomarkers such as IL-6, interferon gamma, and plasminogen activator inhibitor-1. The difference lies in the methodology: hypo- and hyperinflammatory subphenotypes were detected using LCA, whereas the uninflamed and reactive distinction was derived from cluster analyses. Heijnen et al. applied both methods to their cohort [47], each yielding two subgroups with prognostic relevance for mortality. However, the hyperinflammatory and reactive subphenotypes did not fully overlap. Differences in the assignment of subphenotypes between cohorts can have direct clinical consequences, as they can influence prognostic assessment, treatment responsiveness, and the interpretation of stratified clinical trials.

### 4.2. Cardiovascular Subphenotypes

In 2022, Chotalia et al. demonstrated cardiovascular subphenotypes in COVID-19 pneumonitis patients for the first time via echocardiographic and hemodynamic parameters such as the left ventricular end-diastolic area or inferior vena cava diameter [48]. A total of three subphenotypes were detected, which were significantly different in 90-day mortality (22% vs. 42% vs. 73%). Chotalie et al. subsequently identified cardiovascular subphenotypes in an ARDS cohort, using a four-class model [49]. Owing to low separation in the assignment of patients to classes (entropy) but also due to a small number of predictors or a lack of a timeline for performing echocardiography, this subphenotyping was discussed below and must still be evaluated in further cohorts [50]. Nevertheless, the approach also has therapeutic implications, such as guiding ventilation pressures in patients with reduced right ventricular function [51].

### 4.3. Subphenotypes Based on Clinical Routine Data

**Figure 2:**
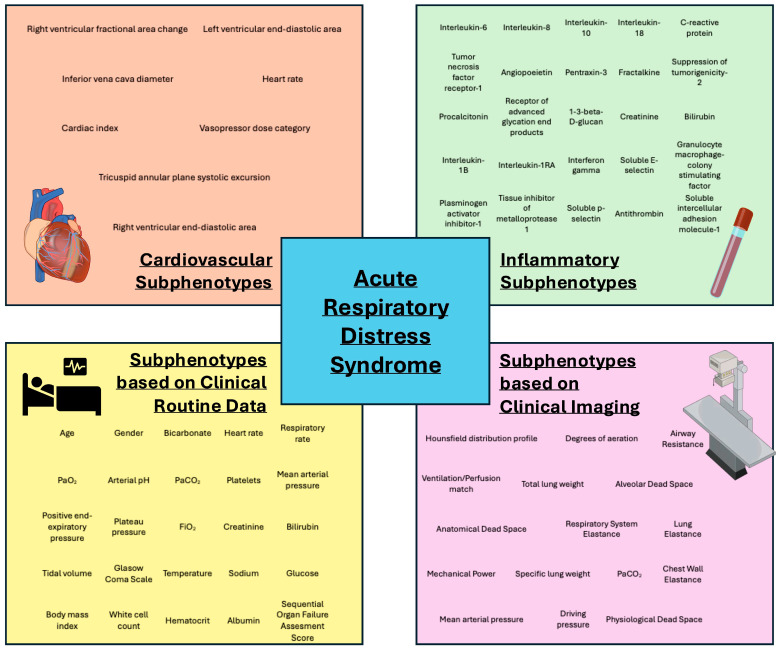
Overview of some of the parameters used in ARDS subphenotyping studies.

Identifying subphenotypes from routinely collected clinical data (Figure 2) offers the advantage of being directly transferable into everyday practice without the need for complex biomarker analyses. Duggal et al. retrospectively analyzed data from six ARDS studies, FACTT [52], EDEN [53], ARMA [35], ALVEOLI [36], SAILS [54] and ART [55], and used K-means clustering to identify two stable subphenotypes on the basis of only nine routine data points, including heart rate, respiratory rate, mean arterial pressure, bilirubin, creatinine, PaO_2_, bicarbonate, pH and FiO_2_ [56]. The subphenotypes showed significant differences in 60-day mortality across five (FACTT EDEN, ARMA, ALVEOLI, and ART) of the six trials, with cluster 2 characterized by higher proinflammatory levels. Due to the aforementioned higher proinflammatory levels, the overlap with previously identified hypo- and hyperinflammatory subphenotypes must be critically discussed. Nevertheless, identifying subphenotypes from readily available clinical data is actually preferable in routine practice compared with more complex biomarker analyses. Sinha et al. [22] and Maddali et al. [57] adopted this approach and presented one (Sinha et al.) and two (Maddali et al.) models, respectively, that classify patients into hypo- or hyperinflammatory subphenotypes based on routine clinical data, such as vital signs and ventilation parameters.

Meza Fuentas et al. identified two subphenotypes based on physiological and ventilation parameters [58], termed ‘efficient’ and ‘restrictive’. The ‘efficient’ subphenotype was characterized by lower compliance (31.08 vs. 40 cm H_2_O/L, *p* < 0.001), lower mechanical power (0.19 vs. 0.24 × 10^−3^ J/min/kg, *p* < 0.001), and lower 28-day mortality (9.3% vs. 21.15%, *p* = 0.021). It is important to note that many of the parameters mentioned, such as mechanical power [59], have already been shown to be associated with mortality. A poorer outcome with more invasive ventilation is therefore to be expected. Nevertheless, the model provides valuable approaches for future studies, such as the response to ventilation maneuvers. Liu et al. were able to demonstrate different responses to therapeutic approaches, such as high or low PEEP or fluid management, using their subphenotyping model [60]. Through retrospective analysis of various ARDS datasets, three subphenotypes were identified. Subphenotype 1 was characterized by low mortality and fewer laboratory abnormalities. Subphenotype 2 correlated with a higher rate of inflammation. Subphenotype 3 correlated with acidosis and renal dysfunction and had the highest mortality rate.

Subphenotypes were also described within specific patient groups. At the beginning of the COVID-19 pandemic, Gattinoni et al. described an L-type (low elastance and low recruitability) and an H-type (high elastance and high recruitability) of COVID-19-associated ARDS [61]. Panwar et al. also applied this approach to pre-COVID-19 ARDS patients [62]. However, this finding was critically discussed and partially relativized as the pandemic progressed [63]. Several further studies have focused on COVID-19-associated ARDS [64,65,66,67,68]. Ceccato et al. analyzed 3743 COVID-19 ARDS patients and identified two clinical subphenotypes in the first three days of intensive care admission using cluster analysis of 44 clinical variables [69]. Evaluation on day one and three revealed a temporal component, with 33% of patients switched from subphenotype 1 to subphenotype 2 and 27% from subphenotype 2 to subphenotype 1, highlighting the importance of the temporal dynamics.

The use of special therapeutic strategies such as prone positioning was also investigated in the context of clinical subphenotypes. Fosset et al. studied 353 ventilated ARDS patients who were placed in the prone position [70]. Cluster analysis revealed three subphenotypes with different ventilation parameters and mortality rates. However, no advantage or disadvantage in the use of prone positioning could be demonstrated. Another focus has been on severe ARDS requiring ECMO. Nishikimi et al. performed a latent class analysis in ECMO patients and identified three subphenotypes—wet, dry, and fibrotic [71]. Notably, 34% of patients were assigned to the fibrotic subphenotype, characterized by fibroproliferative remodeling changes on computed tomography and associated with the highest mortality risk (hazard ratio 1.75; 95% confidence interval 1.10–2.79; *p* = 0.019).

### 4.4. Subphenotyping Based on Clinical Imaging

Imaging provides an important basis for characterizing ARDS subphenotypes. Beyond conventional chest X-ray and computed tomography (CT), functional techniques such as EIT have increasingly been used in recent years to detect heterogeneous patterns of lung involvement [28]. The question of whether the classification into focal and non-focal described above should be considered a subgroup or subphenotype has not been clearly resolved [26]. A key limitation is that radiological findings are often described qualitatively and are difficult to convert into standardized numerical variables. To overcome this limitation, various quantitative scores and indices have been developed in recent years, enabling the reproducible recording of disease severity.

One established approach is the radiographic assessment of lung edema (RALE) score, which quantifies the extent of alveolar edema on chest X-rays. Jabaudon et al. reported that not only the initial RALE score, but especially the dynamic change within the first three days is linked to the prognosis [72]. Another approach is the focal index, derived from Hounsfield units in defined lung regions, which Bjarnadottir et al. used to classify ARDS as focal or non-focal [73]. Combining imaging with biomarkers offers new perspectives for quantification. Partouche et al. examined COVID-19 ARDS patients and assessed the ratio of IL-6 to KL-6 (Krebs von den Lungen) in relation to CT findings [74]. The IL-6/KL-6 ratio showed a linear association with the ground-glass volume in the CT scan.

Wendel Garcia et al. conducted a latent class analysis of ARDS patients, including ventilation parameters and gas volume distribution parameters from CT scans [75]. All CT scans considered were performed under a defined PEEP level. They defined a recruitable subphenotype and a non-recruitable subphenotype, with the recruitable subphenotype being associated with higher mortality (hazard ratio 2.9, 95% confidence interval 1.7–2.7, *p* = 0.001). This classification could also be demonstrated in COVID-19-associated ARDS [76].

## 5. Multi-Omics Approach

**Figure 3:**
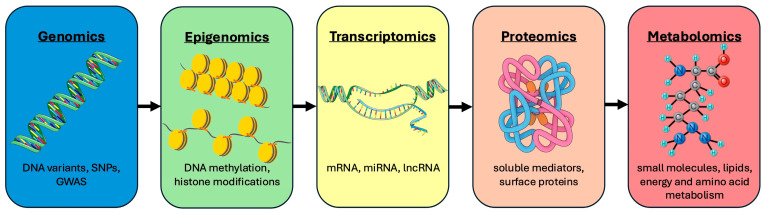
Overview of the multi-omics levels: DNA—deoxyribonucleic acid; GWAS—genome-wide association study; lnc—long non-coding; m—messenger; mi—micro; SNP—single-nucleotide polymorphism; RNA—ribonucleic acid.

Multi-omics refers to the integrated analysis of different molecular levels in order to better understand the complex regulatory and effector mechanisms in ARDS. Although previous multi-omics analyses continue to represent subphenotypes, by examining multiple molecular levels, they enable a much more detailed characterization of the heterogeneity of ARDS, paving the way for the definition of future endotypes. In addition to genomics, this also includes epigenomics, transcriptomics, proteomics, metabolomics, lipidomics [77], analyses of non-coding RNAs, and modern approaches such as single-cell and spatial omics [78]. While all these levels are in principle part of the multi-omics spectrum, genomics, transcriptomics, proteomics, and metabolomics have been the primary focus of research in the context of ARDS to date (Figure 3). However, despite their promise, these approaches remain largely confined to the experimental and translational research domain, with limited feasibility, standardization, and direct applicability in routine clinical care.

With regard to genomics, Xia et al. analyzed existing data sets and identified ten hub genes for distinguishing between high- and low-risk ARDS patients. Of these, six autophagy- and metabolism-related genes (ITGAM, S100A12, HCK, SPI1, PLEK, and MYC) showed different expression levels between the clusters [79]. In addition, twelve infiltrating immune cell types were differentially represented and correlated with the hub genes.

At the epigenomic level, the role of neutrophils in ARDS is being discussed [80]. Two groups of neutrophils have been identified: high-density neutrophils and low-density neutrophils [81]. Low-density neutrophils have been shown to be associated with an increased occurrence of neutrophil extracellular traps [82], which are a fundamental component of ARDS pathogenesis. Heterogeneity of neutrophils in different ARDS etiologies is assumed and may be the target of future therapies [83].

Multiple pathophysiological axes have been described at the transcriptomic level. Analyses of alveolar macrophages revealed that specific transcriptions are directly associated with survival in ARDS [84]. López-Martínez et al. identified two clusters of critically ill COVID-19 patients based on interferon response, with differential outcomes following steroid therapy [85].

Proteomic analyses provide clues to pathophysiological mechanisms and potential biomarkers in ARDS, with most findings derived from preclinical models [86]. Gong et al. identified several differentially expressed proteins in a mouse model and were able to detect six of them—HP, LTA4H, S100A9, SAA1, SAA2, and SERPINA3—in the plasma of COVID-19 ARDS patients [87], showing prognostic value. Chase et al. used club cell secretory protein (CC16) to define patient groups: at a cut-off of 45 ng/mL, those with lower values revealed reduced mortality (7.5% vs. 50%) [88]. However, using the term phenotype for a grouping based on a single biomarker is debatable more accurately reflects a subgroup.

In the clinical field, metabolomics has thus provided the most insights into ARDS subphenotyping. Several studies have demonstrated metabolomic differences between established subgroups and subphenotypes [15,25]. Alipanah-Lechner et al. described the metabolic profile of hyperinflammatory subphenotypes, consisting of elevated concentrations of lactate, pyruvate, and malate with simultaneously reduced circulating lipids [89]. Suber et al. demonstrated differences in the concentrations of acetylcarnitine, octanoylcarnitine, and 3-methylhistidine in the blood of patients with hyperinflammatory and hypoinflammatory ARDS [90]. Rogers et al. examined the pulmonary edema fluid of ARDS patients and patients with hydrostatic pulmonary edema and identified a hypermetabolic subphenotype that was associated with a tendency toward increased mortality (66% vs. 30%) [91]. In addition, Wang et al. characterized lactylation-based phenotypes in sepsis-associated ARDS, identified molecular biomarkers that allow for differentiated risk assessment, and illustrated the influence of metabolic modifications on ARDS pathophysiology [92]. Finally, it should be added that many biomarkers used for inflammatory subphenotyping are also proteins or metabolites and could also be mentioned in this category of multi-omics.

## 6. Endotypes of ARDS

While subphenotypes classify patient groups based on observed clinical or biological characteristics, endotypes focus on specific pathophysiological mechanisms that are causal for the development and progression of the disease. This opens up the door to mechanism-based therapy that extends beyond purely symptomatic approaches. Concepts of endotypes have already been demonstrated in chronic lung diseases, particularly regarding the role of T2 cells [93,94].

No endotypes have yet been developed for ARDS. Although the new multi-omics approach has already revealed complex pathophysiological mechanisms [92], the subphenotypes often lack the causal approach required to be considered endotypes. Therefore, endotypes in ARDS are currently only being discussed theoretically, without any real evidence. Some consider the most well-known subphenotyping in hyperinflammatory conditions to be an inflammatory endotype [95], but here, too, there is also a lack of definitive clarification of the underlying mechanism with the option of causal therapy.

Another proposed endotype is the endothelial endotype [96]. In connection with impaired barrier function and increased vascular permeability, this endotype could also be associated with elevated concentrations of angiopoietin-2 (Ang-2). A meta-analysis revealed that high Ang-2 levels are associated with increased mortality in ARDS patients (odds ratio 1.56; 95% CI: 1.30–1.89) [97]. Prospective cohort studies confirmed that high Ang-2 levels in the early course of the disease are associated with both the development of ARDS and increased mortality [98]. Genetic analyses also suggest a causal role for Ang-2 in ARDS risk [99].

Although no specific endotype based on the renin-angiotensin system (RAS) has been formally defined, endotypes could be considered here. The RAS comprises two opposing signaling pathways: the classic pathway, mediated by angiotensin II via the angiotensin receptor type 1, which triggers vasoconstriction, proinflammatory, and pro-fibrotic effects, and the alternative signaling pathway, which activates the angiotensin receptor type 2 or angiotensin-(1–7) and has vasodilatory, anti-inflammatory, and protective effects [100,101]. Interindividual differences in the activation of these signaling pathways could influence susceptibility to alveolar and endothelial damage in acute respiratory distress syndrome. These observations underscore the importance of individually identifying imbalances between the classical and alternative signaling pathways and, if necessary, addressing them in a targeted manner.

Endotypes in the context of dysregulated immune responses are also conceivable. Studies have shown that low expression of mononuclear human leukocyte antigen DR isotype (HLA-DR) after intensive care treatment and in infection contexts is associated with poor outcomes and an increased risk of secondary nosocomial infections [102]. Such findings have also been comprehensively described in sepsis studies [103]. In a specific pneumonia-related ARDS setting, a significant decrease in HLA-DR expression on circulating and alveolar monocytes was also observed [104]. These constellations suggest that some ARDS patients may have immune paralysis, which could be relevant to infection risks and disease progression.

## 7. Therapeutic Approaches

To date, there is no evidence of treatable traits in the sense of causal, validated therapeutic targets in ARDS. Retrospective analyses show that response to therapy can vary between subphenotypes [105], but these findings have not yet been confirmed in prospective cohort studies or randomized controlled trials. Nevertheless, some evaluations of existing study data provide illustrative examples of how subphenotypes, particularly inflammatory subphenotypes, can respond differently to therapeutic interventions.

A secondary analysis of the HARP-2 study revealed that hyperinflammatory patients treated with simvastatin had significantly lower 28-day mortality than patients treated with a placebo (32% vs. 45%) [106]. This effect was not observed in hypoinflammatory patients (17% vs. 16%). There is also a suspected connection between hyperinflammatory subphenotypes and coagulopathy, which could form the basis of differentiated therapy [107,108].

Additionally, glucocorticoids, conservative fluid management, neuromuscular blocking, inhaled pulmonary vasodilators, individualized ventilation strategies, and recruitment maneuvers are therapeutic measures whose effectiveness is partly subphenotype- or subgroup-dependent [49,51,60,71,75,105,106,109].Using physiological and clinical data, Liao et al. described two ARDS groups that differed not only in mortality but also in response to glucocorticoid therapy [110]. In class 1, there was no significant change in mortality with the use of glucocorticoids, but there was in class 2.

Famous et al. used retrospective data to investigate the effects of liberal and conservative fluid management on two (inflammatory) subphenotypes [109], with subphenotype 2 corresponding to a hyperinflammatory subphenotype. They reported that in subphenotype 1, conservative fluid management was associated with a lower 90-day mortality rate (18% vs. 26%), whereas in subphenotype 2, liberal fluid management yielded a lower mortality rate (40% vs. 50%).

Individualized ventilation strategies, including PEEP optimization and tidal volume adjustment, have similar subphenotype-dependent effects [60,76,111]. In cases of non-focal lung patterns or low compliance, higher PEEP can improve oxygenation, whereas in cases of focal lung patterns, high PEEP can lead to overinflation and increased stress. 

## 8. Current Issues and Future Perspectives

Despite considerable progress in the subphenotyping of ARDS, key challenges remain that currently hinder its translation into clinical routine. A key problem is the lack of consistent criteria for defining subphenotypes. Different studies use different parameters, biomarkers, or methodological approaches, which makes direct comparisons difficult. Realistic identification of differences in therapeutic responses between subphenotypes is only possible on the basis of uniform definitions. 

Furthermore, the practicality and clinical applicability of many analyses must be questioned. While multi-omics approaches provide deeper insights into pathophysiological mechanisms, they are currently hardly applicable in routine clinical practice. In contrast, subphenotypes based on routine parameters offer immediate transferability to patient care, albeit without detailed mechanistic understanding. A combination of both methods, i.e., identifying similar patterns in multi-omics and routine clinical data, should be an important goal in further studies in order to enable routine clinical data to be used as surrogate parameters for more complex pathomechanisms.

A further important aspect is the temporal dynamics of subphenotypes over the course of the disease, which is not reflected in many studies. Even approaches that examine subphenotyping at different points in time and thus demonstrate temporal stability [112] do not use the temporal dimension for phenotyping in the narrower sense. To date, suitable models have been used in only a few studies [113,114].

Another factor that has not yet been fully integrated is the pulmonary microbiome [115], which is increasingly being discussed as a possible influencing factor on subphenotypes and disease progression. Studies have shown that the composition of the pulmonary microflora can influence both the inflammatory response and resistance to secondary infections [116]. Differences in the microbiome may partly explain why patients with similar clinical or inflammatory profiles exhibit divergent disease courses and therapeutic responses [117].

## 9. Conclusions

The phenotyping of ARDS has made significant progress in recent years and provides valuable insights into the heterogeneity of these complex syndromes. Numerous studies have shown that ARDS patients cannot be considered a homogeneous group but that subphenotypes—in particular, the hypo- and hyperinflammatory inflammatory types—are consistently reproducible and have prognostic relevance.

However, ARDS remains a clinical diagnosis based on established criteria. Before subphenotypes or potential endotypes can be discussed as a basis for targeted therapies, there must be consensus on their definition. Different parameters, biomarkers, and methodological approaches currently make comparability and reproducibility difficult. 

To date, endotypes based on clear pathophysiological mechanisms exist only theoretically for ARDS. Although there are indications of inflammatory or endothelial-focused endotypes, their causal mechanisms have not yet been fully clarified.

Until endotypes are identified, it will not be possible to identify treatable traits in a causal sense. Different responses of subphenotypes to therapies have been described in the literature, but still need to be validated by further studies.

Nevertheless, subphenotyping provides a framework for capturing the heterogeneity of the disease, improving patient prognosis, and developing targeted therapeutic approaches. The integration of clinical data, imaging, omics data, and the microbiome will be crucial to identifying robust subphenotypes and potentially causally effective endotypes, paving the way for evidence-based, personalized therapy in ARDS.

## Figures and Tables

**Table 1:** Overview of phenotyping methods.

	Unsupervised Learning (Clustering)	Latent Class Analysis (LCA)	Supervised Learning
Aim	Discovery of new subtypes	Discovery of new subtypes	Assigning new patients to known subtypes
Method	Machine learning (ML)	Statistical modeling methods	Machine learning (ML)
Prior knowledge of subtypes required	No	No	Yes
Procedure	Grouping by similarity	Model-based grouping with probability assignment	Classifier is trained on existing subtypes
Examples	k-Means, hierarchical Clustering	Latent class analysis, latent profile analysis, latent trajectory analysis	Random Forrest, Support Vector Machine
Feature Selection	Optional, ML-based or manual	manual, theory-based	Optional, ML-based or manual
Advantages	Hypothesis-free, data-driven	Model-based, clinically interpretable	directly applicable in clinical practice
Disadvantages	Interpretation sometimes difficult	less flexible	Dependent on a good training basis, only for known subtypes

## Abbreviations

The following abbreviations are used in this manuscript:

Ang-2	Angiopoietin-2
ARDS	Acute respiratory distress syndrome
CT	Computertomography
ECMO	Extracorporeal membrane oxygenation
EIT	Electrical impedance tomography
HLA-DR	Human leukocyte antigen DR isotype
IL	Interleukin
KL	Krebs von den Lungen
LCA	Latent class analysis
ML	Machine learning
PEEP	Positive end-expiratory pressure
RALE	Radiographic assessment of lung edema
